# History of trauma and associated symptoms in adolescent and young adult prison population

**DOI:** 10.1192/j.eurpsy.2026.11288

**Published:** 2026-07-31

**Authors:** P. Canut Montes, A. Armendariz, Y. Sobrino, N. Del Prado

**Affiliations:** Parc Sanitari Sant Joan de Deu, Sant Boi de Llobregat, Spain

## Abstract

**Introduction:**

A history of traumatic experiences in childhood and adolescence has been consistently linked to an increased risk of developing mental health problems and maladaptive behaviors in adulthood. The prison population constitutes a particularly vulnerable group due to the high prevalence of a history of trauma and the presence of associated symptoms, such as affective disorders, anxiety disorders, and dissociative symptoms. Understanding the relationship between trauma and its clinical impact on young inmates is essential for guiding preventive and early intervention strategies.

**Objectives:**

To determine the prevalence of a history of traumatic experiences in the adolescent and young adult prison population, describe the most common types of trauma, analyze the associated clinical symptoms, and explore their relationship with sociodemographic variables.

**Methods:**

Single-center, observational, cross-sectional study based on questionnaires administered to adult patients admitted to the Can Llupià, Alzina, and Til·lers Educational Centers between September 2023 and April 2025. An analysis of the collected data was carried out, focusing on the history of traumatic experiences and the presence of associated clinical symptoms (anxiety, depression, dissociation, among others), as well as other sociodemographic variables.

**Results:**

The sample included 30 patients, 96% of whom were male. Eighty-three percent reported at least one traumatic event, with a common accumulation of traumatic experiences (more than half reporting three or more). The first trauma usually occurred between the ages of 8 and 15. The most common were domestic violence (36%), other physical assaults (12%), and the unexpected death of a family member or friend (8%); 28% reported experiences not captured in the questionnaire. Furthermore, 64% had experienced migration, half of them alone.

Regarding associated clinical symptoms, the most prevalent symptoms were insomnia (76%), nightmares (72%), concentration problems (68%), and hypervigilance (64%). Flashbacks (64%) and emotional disconnection (64%) were also common. Overall, 20% of the sample met diagnostic criteria for PTSD.

**Conclusions:**

The high prevalence of traumatic experiences and associated symptoms in this population highlights the need for systematic screening and early intervention in juvenile detention centers. The relationship between traumatic experiences and the development of criminal behavior constitutes a priority line of research, which could contribute to the design of more effective preventive strategies and improve both clinical prognosis and social reintegration.

**Disclosure of Interest:**

None Declared

